# Radial rotation of cell-pair under beam mode coupling effect of microcavity cascaded single fiber optical tweezers

**DOI:** 10.1515/nanoph-2025-0033

**Published:** 2025-03-31

**Authors:** Zhaoqi Ji, Chunlei Jiang, Peng Chen, Linzhi Yao, Minghui Zhang, Qizan Shi, Cun Zhao, Xiufang Wang, Yu Sun, Taiji Dong

**Affiliations:** 117792 Northeast Petroleum University, 199 Fazhan Road, Daqing, China

**Keywords:** microcavity, beam mode coupling, cell-pair rotation, single fiber optical tweezers

## Abstract

This article presents a control method for radial cell-pair rotations using a single-fiber manipulation technique that combines microcavity cascade optical tweezers with optical fiber mode coupling technology. It explores the mechanisms of cell manipulation under the influence of mode coupling and capillary fluid forces. By controlling the angle of fiber twisting and utilizing the birefringence effect along with the principle of beam mode coupling, it is possible to achieve precise and regular variations in the energy of the LP21 mode beam spot, thereby altering the magnitude and direction of the forces acting on the cell-pair, which induces a tendency for rotational motion. The microcavity cascade optical tweezers provide a small capillary fluid force and serve to isolate the cell-pair from the external environment, allowing it to respond to changes in beam spot energy within a stable microcavity space, thus enabling controllable rotations in both direction and angle. The combination of microcavity cascade optical tweezers with beam mode coupling technology achieves, for the first time, radial cell-pair rotations driven by a single fiber, which holds significant implications for the study of polarized cell migration as well as the investigation of tissue fluidity and connectivity dynamics in cancer prediction.

## 1 Introduction

Cell rotation plays a pivotal role in biological research, profoundly influencing the morphology and function of cells, while also playing an important role in the processes of cell migration and differentiation. By altering the interactions both within and outside the cell, cell rotation can affect the structure and shape of tissues; influence intercellular signal transduction, nutrient exchange, and cellular coordination; as well as enhance sensitivity to external stimuli, particularly during development [1], [2], [3] and tissue repair [4], [5], [6]. Investigating the mechanisms of cell rotation contributes to a better understanding of the fundamental principles of cell biology [7], [8] and provides important technical methods for developing treatment strategies for related diseases [9].

Optical tweezers technology is a technique that utilizes highly focused beams of light to achieve precise, non-invasive manipulation of microscopic objects. Optical field modulation technology refers to the effective control of the fundamental parameters of the optical field, including frequency, amplitude, phase, and polarization [10], [11], [12], [13]. These technologies have found widespread application in fields such as acoustics, interfacial science, biology, and biophysics [14], [15], [16], [17], [18], [19]. By combining optical tweezers technology with optical field modulation technology, significant advancements have been made in the manipulation of cell rotation. For example, optically driven cellular spins through circularly polarized light [20] face limitations as they cannot achieve cell-pair rotations and can only induce continuous rotations around the origin of the cells. Additionally, Black et al. demonstrated a method using dual optical fibers to effectuate the inversion of two cells [21], but this method requires the dual fibers to remain in the same plane, leading to complexity in operation and confinement to a small operational area. Holographic optical tweezers can also facilitate multi-beam cell rotations [22], yet this technique is confined to longitudinal control for cell-pair rotations and has not achieved breakthroughs in radial manipulation. Moreover, complex special optical tweezers combined with multiple physical fields have enabled cell rotations, as evidenced by N. Hameed et al., who utilized a silver-coated and nanopore port single optical tweezers to achieve cellular manipulation [23]. Sun et al. proposed a method for cellular control through the combined effects of a temperature gradient and PEG concentration [24]. However, the aforementioned technology has drawbacks such as complex manufacturing processes, high operational difficulty, and a tendency to cause thermal damage to cells, as well as an inability to control the rotational position and angle of the cells.

Therefore, utilizing a compact and easy-to-operate single-fiber optical tweezer to achieve controllable and stable radial rotations of cell pairs holds significant importance and research value. In this regard, we propose a novel control method for cell-pair rotations based on a cascaded microcavity optical tweezer. We will conduct an in-depth study of the theory of fiber mode coupling and the mechanisms combining optical forces with microcavity capillary fluid dynamics, exploring the impact of fiber twisting techniques on the energy distribution of LP21 mode beams and the influence of beam mode coupling on cell manipulation. Moreover, through the isolating effect of microcavity optical tweezers on the external environment, cells can respond to changes in the energy of light spots within a stable microcavity space. This has enabled, for the first time, precise control over radial cell-pair rotations and their rotational angles under single-fiber actuation. In this process, the controllable rotation of cells has been concretely realized, and the cellular dynamics mechanisms arising from the coupling of beam modes and capillary fluid forces have been clarified, demonstrating the potential applications of optical technology in cellular dynamics.

## 2 Methods

### 2.1 Theory

This article investigates the mechanism of cell manipulation under the combined effects of optical fiber mode coupling and capillary fluid dynamics, as well as the phenomenon of cell-pair rotations. To achieve this, it is necessary to first excite higher-order mode beams within the optical fiber. The principle of higher-order mode beam excitation is as follows: when the transmission wavelength exceeds the cutoff wavelength, only the fundamental mode LP01 exists in a single-mode fiber; conversely, when the transmission wavelength is below the cutoff wavelength, other lower-order or higher-order mode beams will be correspondingly excited in the single-mode fiber. The number of modes propagating in the fiber depends on the normalized frequency parameter *V* for the transmitted light wave within the fiber: when *V* < 2.405, only the LP01 mode beam can propagate; when 2.405 ≤ *V* ≤ 3.832, the higher-order LP11 mode beam will be generated and propagate; and when *V* > 3.832, the LP21 mode appears. The formula for calculating the normalized frequency parameter *V* [25] is as follows:

(1) $$V=\frac{2\pi a}{\lambda }\sqrt{n_{1}^{2}-n_{2}^{2}}$$

In this context, *λ* represents the wavelength of the laser; a is the core radius of the single-mode optical fiber; *n*_1_ is the refractive index of the core; and *n*_2_ is the refractive index of the cladding. Using a standard single-mode optical fiber with a wavelength of 1,550 nm, the core radius a is 4.5 µm, with a core refractive index of *n*_1_ = 1.467 and a cladding refractive index of *n*_2_ = 1.459. When the incident laser wavelength *λ* is 650 nm, it can be deduced that *V* ≈ 6.655 > 3.832, which indicates the presence of the LP21 mode in the fiber. As a result, various mode spots can be observed, effectively making this fiber function as a multimode fiber. We spliced the mismatch 5 µm single-mode fiber [26], at this time, the LP21 mode accounted for a high proportion and the effect was good, and the mode coupling mainly occurred between LP11 and LP21 modes.

In a microcavity, optical force analysis can be conducted using the Maxwell stress tensor method. The time-averaged Maxwell stress tensor is typically denoted by <*T*_M_>. In an electromagnetic field, it describes the average stress exerted by the electromagnetic field on the medium. When the integration is performed over a closed surface *S* surrounding the target particle, *n* is the outward unit vector normal to *S*. The total optical force *F* applied to the cell can thus be expressed as:

(2) $$F=\underset{S}{\int }\left(\left\langle T_{M}\right\rangle \cdot n\right)dS$$

At the same time, the presence of capillary phenomena can induce a certain flow velocity of the liquid, thereby exciting the Stokes drag force *F*_D_ on the particles, which can be defined by Stokes’ law and the velocity response time *τ*_p_ of spherical particles.

(3) $$F_{D}=\frac{m_{p}}{\tau_{p}}\left(u-v\right),\tau_{p}=\frac{\rho d^{2}}{18\mu }$$

*u* and *v* represent the velocity of the fluid and the velocity of the particles, respectively. *m*_p_ is the mass of the particles, while *ρ* and *d* refer to the density and diameter of the particles, respectively. *μ* denotes the dynamic viscosity of the fluid.

Therefore, the resultant force *f* acting on a single cell can be expressed as: *f* = *F* + *F*_D_.

The LP21 mode is equivalent to the symmetric superposition of two LP11 modes; therefore, for circular core fibers, it suffices to discuss either the even or the odd mode of LP21. Taking the even mode of LP21 as an example, by selecting an appropriate working wavelength for the light source, both the LP11 mode and the LP21 even mode can be transmitted within the fiber core. The superposition of these two modes results in an interference field at the fiber output end, with the interference output light intensity being [27]:

(4) $$\begin{array}{ll} I & ={\left|E\left(x,y\right)\right|}^{2}=E_{11}^{2}\left(x,y\right)+E_{21}^{2}\left(x,y\right)\\ & \;+2E_{11}\left(x,y\right)E_{21}\left(x,y\right)\cos\left(\Delta \varphi \right) \end{array}$$

In the equation, Δ*φ* represents the phase difference between the LP11 mode and the LP21 even mode after transmission through the optical fiber, where Δ*φ* = Δ*β* × Δ*L*. Here, Δ*β* denotes the difference in propagation constants between the LP11 mode and the LP21 even mode, expressed as Δ*β* = *β*01 − *β*11, and Δ*L* signifies the change in the length of the optical fiber. *E*_11_ and *E*_21_ are the mode field distributions for the LP11 mode and the LP21 even mode, respectively. When the optical fiber is subjected to external disturbances, the intensity of the interference light output from the fiber varies with changes in the intermodal phase difference, manifesting as an energy exchange in the output side lobes of the two coupled modes.

### 2.2 Design of device

**Figure 1:**
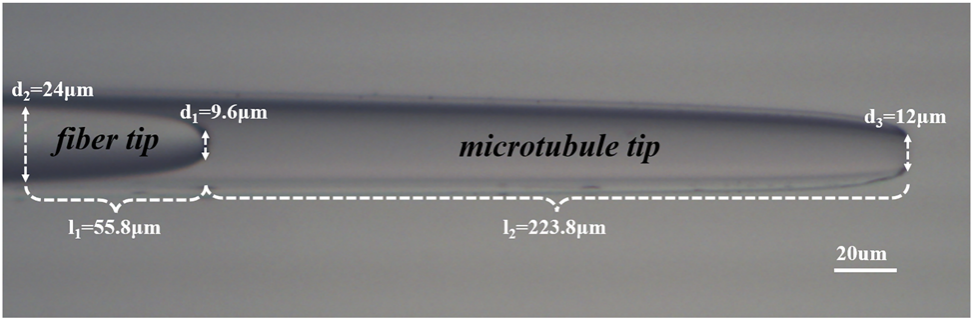
Physical image of the cascaded microcavity optical tweezers probe.

We employed a cascaded microcavity structure that combines a single-mode optical fiber with a capillary microtube. Initially, a commercial SMF optical fiber (connector type FC/PC, core diameter 9 µm, cladding diameter 125 µm, Corning Inc.) was subjected to a heating and stretching technique to form a specific fiber tip. After the fiber was slightly melted, it was stretched at a speed of 0.5 m/s until the fiber tip length was reduced to l_1 _= 55.8 µm, and a tapered flat tip of d_1 _= 9.6 µm was formed, shaped by the surface tension of the molten fiber. The method of making the tip of the microtube is the same as that of the tip of the fiber, and the hollow microtube with inner diameter d_2 _= 24 µm, port d_3 _= 12 µm and length l_2 _= 223.8 µm (micro-cavity optical waveguide) is finally formed as shown in Figure 1.

**Figure 2:**
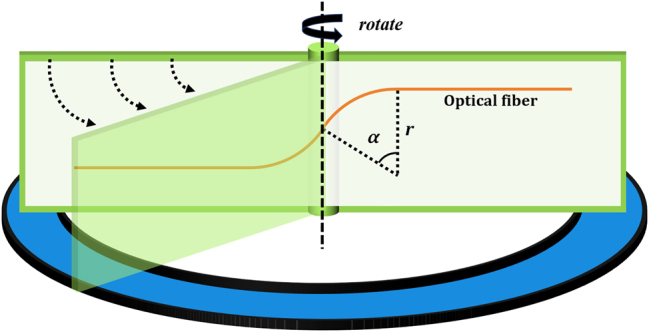
Schematic diagram of the optical fiber twisting shrapnel device.

The optical fiber controllable twisting device and optical fiber shrapnel as shown in Figure 2 are made, and the optical fiber is bent to form two opposite arcs, while the curvature radius is tangentially intersected, and the optical fibers at both ends of the arc are extended vertically and parallel. In this paper, suitable dimensions are selected, specifically *r* = 1 cm and a circular arc of *α* measuring 40° [28]. By rotating the optical fiber spring model, it becomes possible to achieve a fixed controllable angle of twist on the optical fiber.

**Figure 3:**
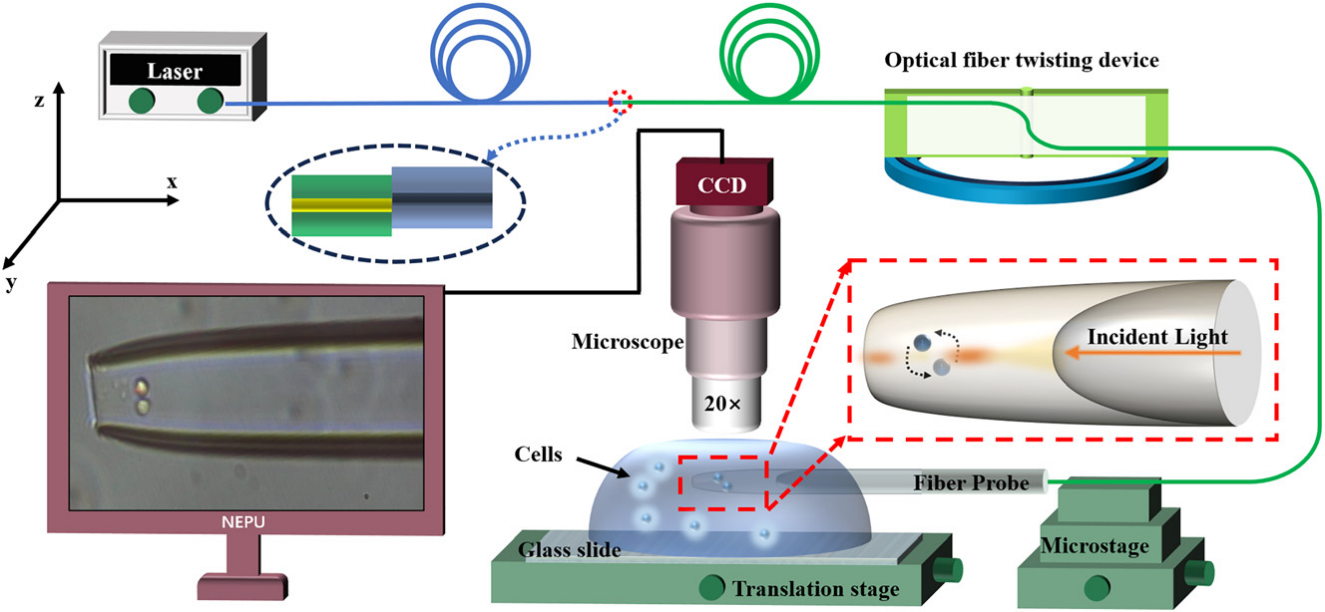
A schematic diagram of the experimental setup.

To achieve the capture and manipulation of cells (cell-pair rotations) and to investigate their relationship with higher-order mode coupling, we designed an experimental apparatus, as shown in Figure 3. In the experiment, a laser (YA605, nbyebo) with a wavelength of 650 nm and a power of 25 mW was used as the light source. We employed a yeast solution with a diameter of 5 μm as the experimental sample; this was accomplished by splicing a 650 nm single-mode fiber with a 1,550 nm single-mode fiber with a mismatched overlap of 5 μm to form an LP21 mode. Additionally, the optical fiber probe tip of the cascaded microcavity optical tweezers was first placed in the solution and allowed to stand for 10 min to eliminate bubbles, and it was connected to a microscope equipped with a CCD camera to record experimental images.

## 3 Results

In this paper, an optical fiber twisting device will be used to conduct experiments on cell-pair rotations with cascades of microcavities as the carrier and optical tweezers as the capture means, proving that the optical fiber twisting can achieve controllable changes in the light field. The 650 nm single-mode fiber and 1,550 nm single-mode fiber mis-mode 5 μm welding, through the input of 650 nm wavelength constant power laser, can stimulate the LP21 beam at the fiber tip, and design a special conical fiber tip for converging beam, generate a strong gradient force near the fiber tip, combined with the capillary force in the microcavity, Cells can then be stably trapped in microtubules. Then, the optical fiber is twisted at a fixed angular speed to promote the energy transfer of the four light spots of the LP21 mode, so as to realize the mixing and switching of the modes, the beam will be changed from the LP11 mode to the LP21 mode and then to the LP11 mode. Therefore, the cell pair will be controlled by different light fields and carry out radial rotation with controllable Angle.

**Figure 4:**
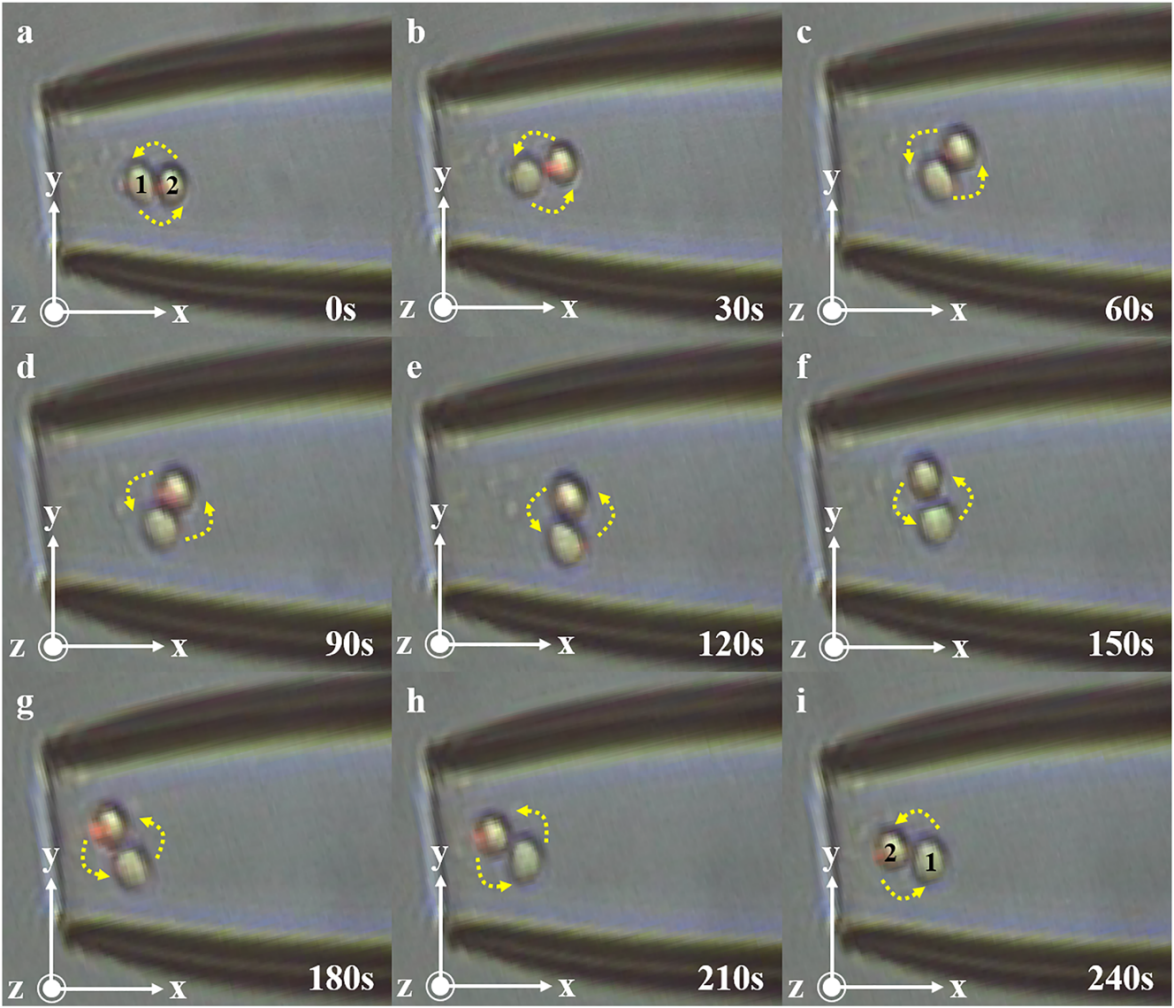
Schematic representation of 0–240 s cell pair rotation. (a) At 0 s, cell 1 and cell 2 in the cell pair were in the horizontal position. (b) At 30 s, cell 1 and cell 2 in the cell pair began to rotate counterclockwise. (c) At 60 s, the cell pair rotated about 45°. (d) At 90 s, the cell pair continued to rotate. (e) At 120 s, cell 1 and cell 2 in the cell pair were in a vertical state. (f) At 150 s, the cell pair was further rotated. (g) At 180 s, the cell pair continued to rotate. (h) At 210 s, the cell pair continued to rotate. (i) At 240 s, cells 1 and 2 in the cell pair return to the horizontal state, but their positions are switched.

As shown in Figure 4, the initial distortion Angle is 0°, and cells 1 and 2 are stably captured in the microcavity, as shown in Figure 4a, cell 1 on the left and cell 2 on the right. When the fiber is slowly rotated at 0.125°/s, cell 1 gradually moves to the right, cell 2 moves to the left, and finally at 240 s, cell pair rotations are achieved.

**Figure 5:**
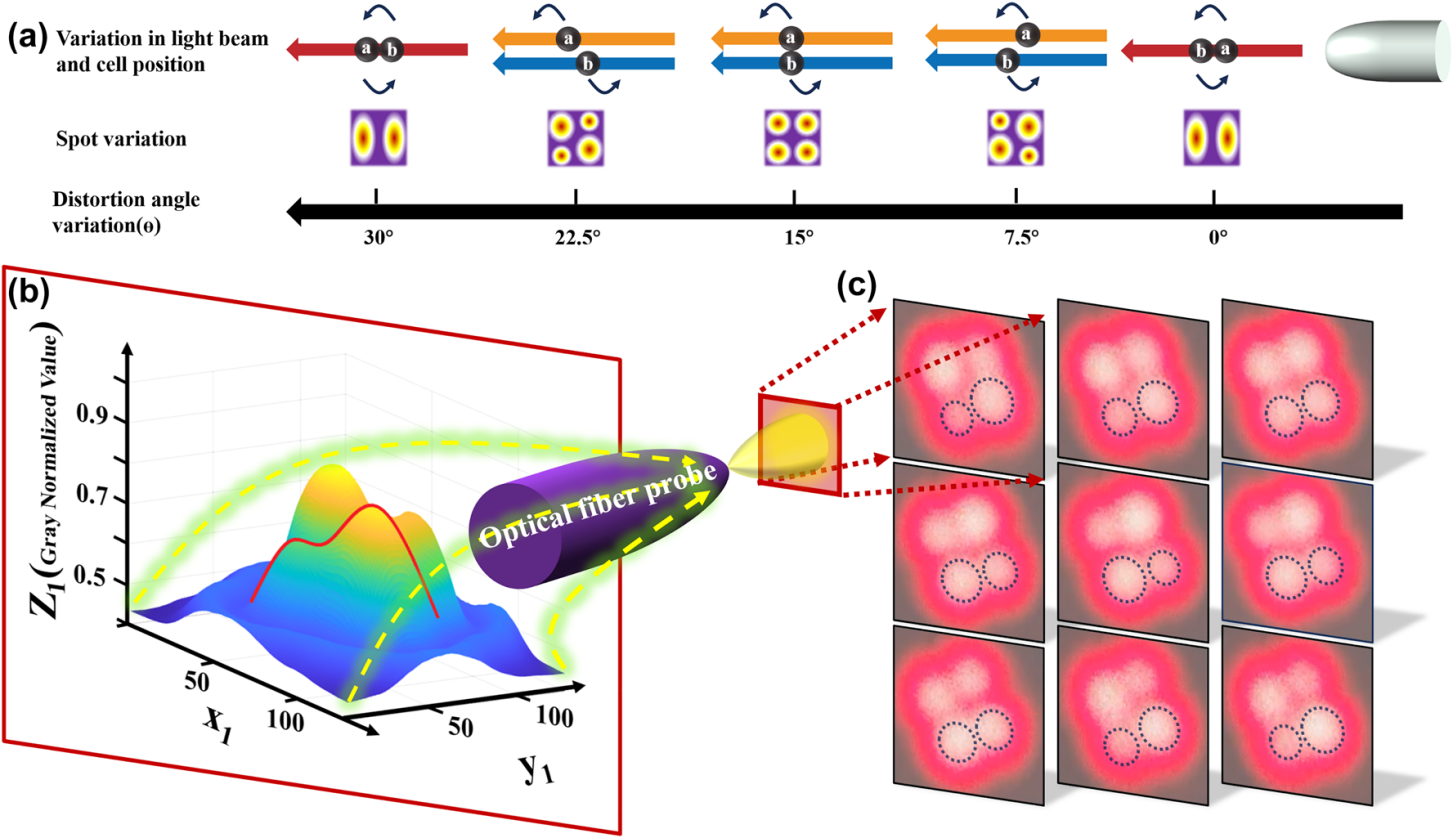
Schematic representation of the mode-coupled transformation of the LP21 mode beam. (a) Schematic diagram of the variation of light spots and cells with rotation angle. (b) Light spot input diagram. (c) Diagram of the light spot changes captured by CCD.

The transfer of spot energy and the change of beam mode are realized by twisting the fiber. In combination with micro-cavity optical tweezers, the process of cell-pair rotations can be controlled. Then, based on the experimental results, a reasonable guess was made, as shown in Figure 5a, to further study the principle of fiber mode coupling and the cell manipulation mechanism under the combined action of fiber mode coupling and capillary force.

When the initial distortion of the fiber is 0°, the laser presentation is dominated by the approximate LP11 mode (imperfect LP11), and its energy is concentrated in two separate spots, and the cell will move in one of the spots. At this point, if LP11 is regarded as the LP21 mode, two spots of LP21 mode can be seen in one of the spots of LP11 (the energy between the two spots is distributed according to the ratio of 7:1), resulting in the affected cell A and cell B arranged in A single column along the axial direction. Cell B is trapped behind by the refocusing effect of cell A on the beam. As the fiber distortion gradually increased to 7.5°, the LP11 mode began to transition to the LP21 mode, and the energy distribution showed a dynamic evolution: the main spot energy gradually split from the initial 8 units into two sub-spots (6:2→5:3→4:4). During this process, spot 1 with attenuated energy drives cell B to move forward, while spot 2 with enhanced energy drives cell A to move backward. At the same time, the transverse division of the spot initiates a dimensional transformation of the cell arrangement, and the single-column structure gradually differentiates into a dual-row rudiment with longitudinal spacing. When the distortion reached the critical value of 15°, the system completed the complete construction of LP21 mode, forming four energy balanced spots (4:4). At this time, the cells presented A typical double-row two-dimensional arrangement: cell A was stable in the upper position under the action of the upper spot, and cell B was positioned in the lower position under the control of the lower spot, and the two formed a vertical spatial separation. As the degree of distortion continued to increase to 30°, the mode of the system reversed, and LP21 refused to LP11 mode. At this stage, the spot energy gradually migrates from the 4:4 of the double column distribution to the extreme distribution of 1:7, resulting in backward movement of the cell - cell A continues to move backward as the end spot energy increases, while cell B moves forward as the front spot energy decreases. Finally, when the two spots were completely merged, the cells returned to A single-row axial arrangement, but the spatial order was reversed: cell A was locked at the rear end, and cell B was fixed at the front end, thus achieving a complete 180° controllable rotation regulation. The process realizes the reversible conversion of the light field mode (LP11↔LP21) by precisely regulating the fiber distortion, and then the three-dimensional reconstruction of the cell spatial arrangement is carried out. To validate the hypothesis regarding the cell manipulation mechanism under the combined effects of optical fiber mode coupling and capillary fluid dynamics, it is necessary to verify the changes in beam mode after twisting the optical fiber, resulting in the transformation of a four-lobed light spot into a two-lobed light spot. Simulations have already been conducted on the power fluctuations of the microcavity-coupled optical tweezers, confirming that changes in power lead to shifts in the position of the trapping wells within the microcavity system, thereby enabling cell-pair rotations.

In the experiment, a CCD camera was used as the means for image acquisition, and a projection method was employed to capture the changes in the light spot. The projection method involves projecting the light spot emitted by the optical fiber vertically onto a smooth, uniform, and appropriately colored (reflective) flat surface. A nearby macro camera continuously captures the changes in the light spot. The data collected by the CCD is in the form of video, which requires image processing. The actual image acquisition data is shown in Figure 5c.

**Figure 6:**
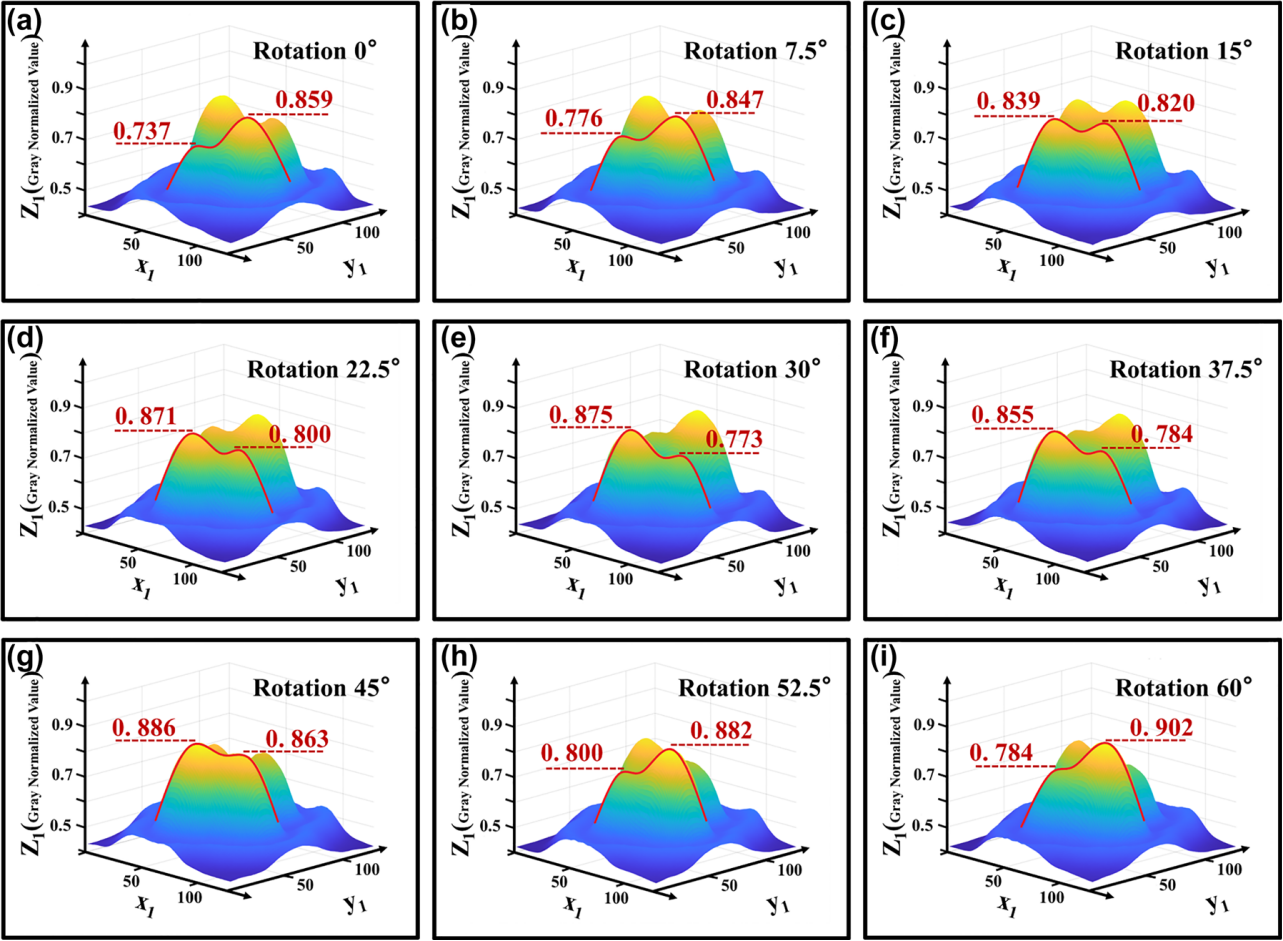
3D light spot distribution after normalized gray smoothing. (a) The normalized intensity of one pair of spots of LP21 was 0.737 and 0.859, respectively, when the fiber twisting device was 0°. (b) When the fiber twisting device was 7.5°, the energy transfer of a pair of spots of LP21 began. (c) When the fiber twisting device was 15°, the normalized intensity of one pair of spots of LP21 was 0.839 and 0.820, respectively, which was approximately equal. (d) When the fiber twist device was 22.5°, a pair of LP21 spots continued to transmit energy. (e) When the fiber twisting device is 30°, the normalized intensity of one pair of spots of LP21 is 0.875 and 0.773, respectively, which is opposite to the situation in (a). When the fiber twisting device is 45°, the situation is similar to that in (c). (f–h) When the fiber was twisted from 37.5° to 52.5°, the energy of a pair of LP21 spots started to transfer in the opposite direction. (i) Similar to (a) when the fiber twisting device is 60°.

Next, the image data is processed to convert it to greyscale and to present it in a three-dimensional format, allowing for an analysis of the variations in greyscale values. The steps involved include graying and compression, followed by uniformity adjustments of the light spots. The processed image is shown in Figure 6. Subsequently, the center points of two light spots are identified along with their specific greyscale values, with the data presented in Table 1.

**Table 1:** Normalized gray values and difference between left and right spots after twist fixed angle.

Angle of rotation	Left spot	Right spot	D-value
0°	0.737	0.859	−0.112
7.5°	0.776	0.847	−0.071
15°	0.839	0.820	0.019
22.5°	0.871	0.800	0.071
30°	0.875	0.773	0.102
37.5°	0.855	0.784	0.071
45°	0.886	0.863	0.023
52.5°	0.800	0.882	−0.082
60°	0.784	0.902	−0.118

**Figure 7:**
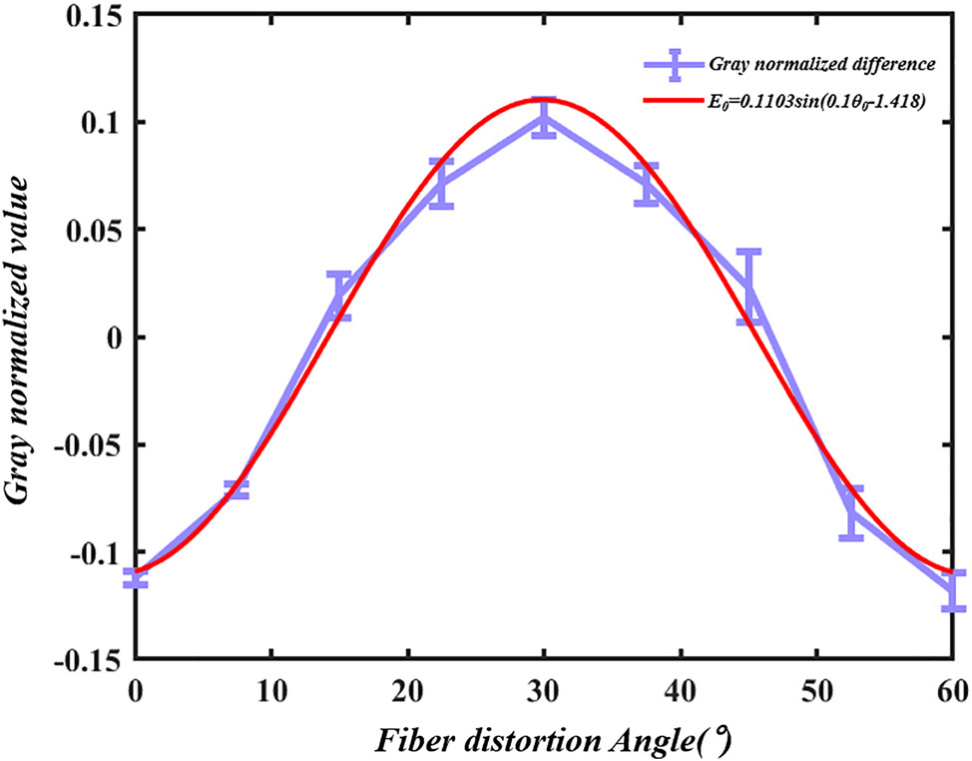
Diagram of the relationship between the grayscale value differences of light spots and the rotation angle.

Experimental results indicate that the energy of the light spot undergoes systematic migration. By treating the LP21 mode as a symmetrical LP11 mode, we can take half of the LP21 as an example. In the initial state, the light spot energy is characterized by one high and one low value, corresponding to a large and a small spot, with the small spot attached to the large spot, which can be regarded as a single light spot. At this moment, the beam can be approximated as the LP11 mode. When rotated by 15°, the energy ratio approximates to one-to-one, resulting in a clear separation between the two spots, thus forming a perfect LP21 mode. Upon a further rotation of 30°, the energy continues to migrate, altering the energy distribution to one low and one high, splitting back into one small and one large spot, which can once again be seen as a single light spot, returning the beam to the LP11 mode. Experiments have demonstrated that, when an optical fiber is twisted at a certain angle, the energy of the optical spot varies accordingly. At the same time, the changes in the light spot exhibit a cyclical pattern: as shown in Figure 7, the difference between the two light spots has a periodicity of 30 degrees of distortion. According to the data in Table 1, the corresponding curve between the difference of normalized gray value and the fiber distortion Angle can be fitted as follows:

(5) $$E_{0}=0.1103\sin\left(0.1\theta_{0}-1.418\right)$$

where *E*_0_ is the difference between the normalized gray values of the left and right spots, and *θ*_0_ is the fiber distortion angle.

## 4 Analysis of simulation

**Figure 8:**
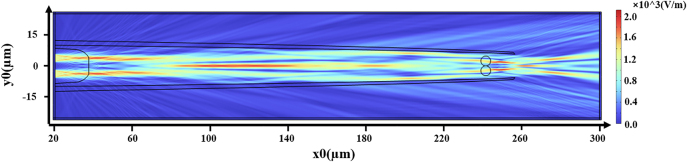
Schematic diagram of COMSOL simulated light field.

In this paper, to analyze the focusing light field characteristics of composite mode optical fibers, a two-dimensional model was established using the electromagnetic wave frequency domain module in COMSOL simulation software, based on the finite element method. The size of the grid was set to be 1/5 of the wavelength of the incident laser. The simulation conditions are as follows: the wavelength of the light source is 650 nm, and the radius of the optical fiber core is 4.5 µm. The simulation model was designed completely according to the real object. The material of the fiber and the microcavity was silica, the solution was water, and the refractive index was 1.46 and 1.33, respectively. And the outermost layer is set to the perfect matching layer. The diameter of the cells is approximately 5 µm, with a refractive index of about 1.39. The upper and lower laser spots are set to 5mW respectively. The simulation results are presented in Figure 8, calculating the optical radiation pressure on the particles.

**Figure 9:**
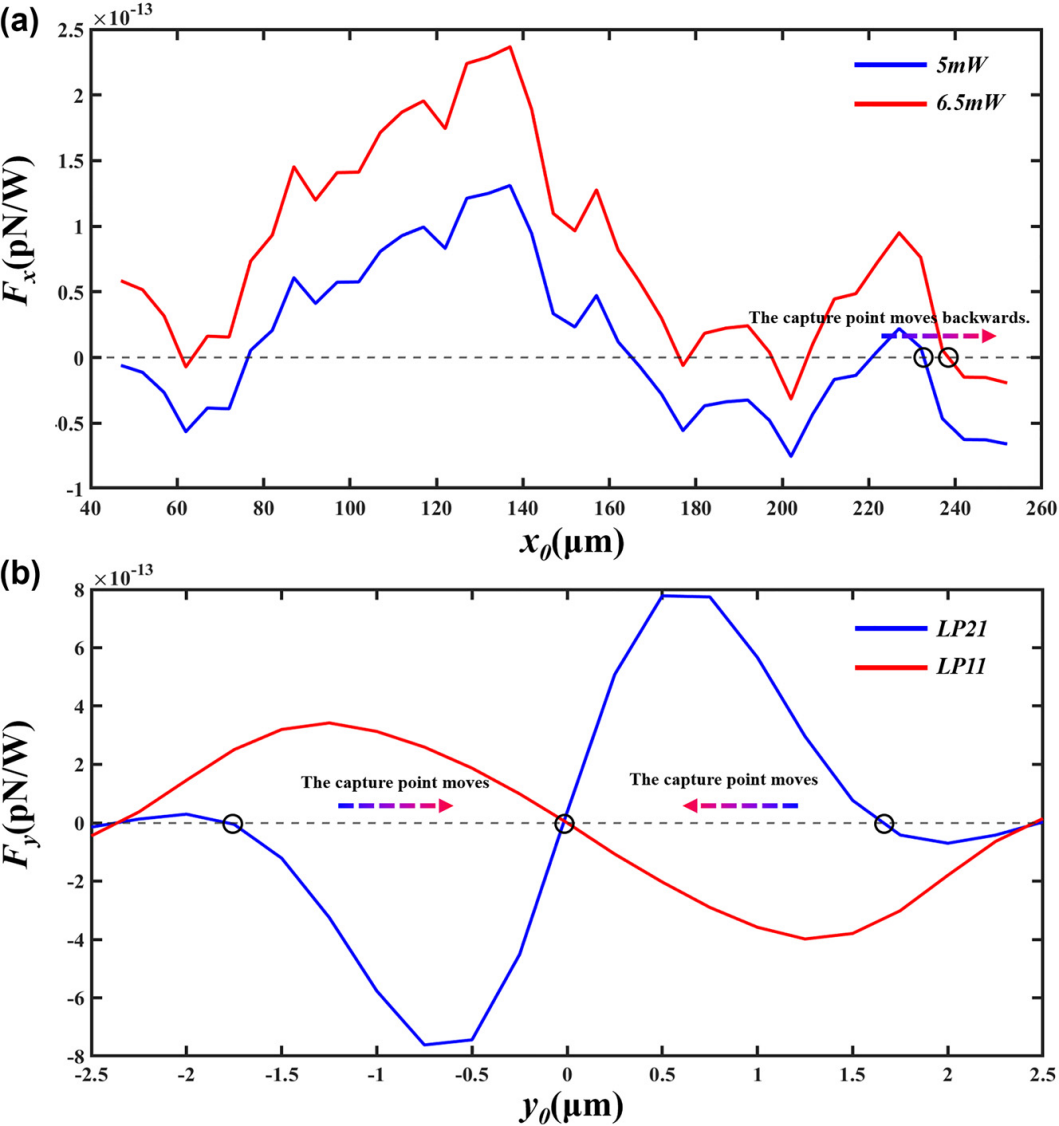
Schematic representation of cell stress. (a) Longitudinal stress curve of a cell. (b) Radial stress curve of a cell.

In this experiment, the movement of cells is influenced by capillary fluid forces. The speed of movement can be derived from measuring the distance and duration of the cells entering a microtube in a light-free environment. Applying Stokes’ law, the capillary fluid force of the apparatus is calculated to be 2.10e–13 N, directed along the *x*-axis towards the origin. The simulation results of the force curve on the cells in the longitudinal direction are shown in Figure 9a. Variations in the input power of the optical tweezers along the *x*-axis direction will cause shifts in the trapping point. The blue line shows the force curve of cells with both input ports at a power of 5 mW, with the capture point stabilized at *x* = +235 µm. The red line displays the force curve of cells with both input powers at 6.5 mW, with the capture point stabilized at *x* = +239 µm. Conclusion: In the microcavity cascade optical tweezers system, increasing beam power results in the trapping point moving backwards, while a decrease in power causes the trapping point to advance.

The cell was determined to be located at *x* = +235 µm, and the radial force curve of the cell was simulated. As shown in Figure 9b, the red line in the figure represents the radial force curve of the cell in the LP11 mode, while the blue line represents the radial force curve of the cell in the LP21 mode. According to the conclusion of the coupling between spot patterns, when the two spots of the LP21 mode gradually approach and merge to become the LP11 mode, the cell will stabilize at the position of *y* = 0 μm; when one spot of the LP11 mode gradually splits to form two spots of the LP21 mode, the cell will stabilize at the position of *y* = ±1.7 μm.

**Figure 10:**
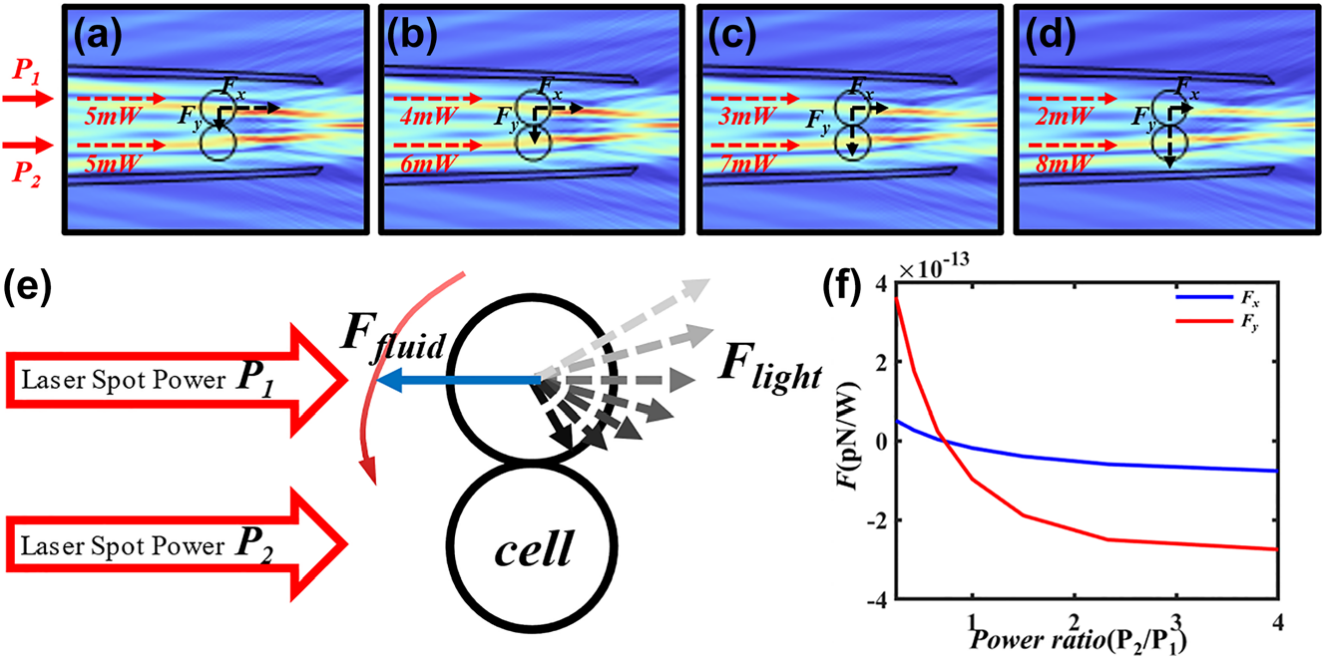
Rotation analysis of cell pairs. (a)–(d) Simulation diagram of laser spot power ratio change, (e) diagram of cell rotation trends, (f) the corresponding curve of cell force and power ratio in *x* direction and *y* direction.

We investigate the tendency of cells to move in response to rotation by varying the power of different laser spots in the input. Taking one of the cells as the subject for force analysis, we explore the trend of its optical force changes. As shown in Figure 10, the input power of light spot 1 is P_1_, and the input power of light spot 2 is P_2_. By adjusting the simulation powers P_1_ and P_2_, we found that, as illustrated in Figure 10f, the light forces acting on the upper cell in the *x*-axis direction and the *y*-axis direction are inversely proportional to P_2_/P_1_. Specifically, when the powers P_1_ and P_2_ vary as depicted from Figure 10a–d, the resultant direction of the light force acting on the cell changes as shown in Figure 10e. The direction of the flight will point diagonally behind the other cell in the cell-pair, and combined with the fluid force fluid, the cell will obtain the movement trend shown by the red curve in Figure 10e. Similarly, based on symmetry, one cell can exhibit a tendency for rotational movement in the opposite direction relative to another cell. Through this simulation and data analysis, the phenomenon of cell-pair rotation observed in experiments can be confirmed.

The light spots transform from a single entity into two and then back into one as the degree of distortion changes. Therefore, by twisting the optical fiber, one can achieve the merging and splitting of the optical spot, as well as a stable distribution of its energy. It is foreseeable that, with certain operations, controllable coupling of beam modes can be realized, and this method allows for easy switching of beam modes and enables controlled inversion and rotation of cells.

## 5 Conclusion and discussion

In our experiments, we observed intrinsic spinning behaviour of particles during rotational manipulation of cell pairs, which may be attributed to frictional interactions arising from cell surface roughness. Concurrently, we detected a probabilistic occurrence of near-contact adhesion between cells during rotation. These phenomena warrant further investigation and may be elucidated through future studies.

This experiment and simulation clarified the mechanism of cell movement under the combined action of fiber mode coupling and capillary force. By using the Angle of the twisted fiber, the spot energy transfer of the LP21 mode can be effectively realized, and then the mode conversion can be realized. At the same time, the change of optical power in the cascade microcavity optical tweezers combined with the influence of capillary flow force can realize the advance and regression of the particle stable trapping point. In this experimental simulation, the laser power increases from 5 mW to 6.5 mW, and the cell position moves backward. The change of spot energy (that is, the change of laser power) and the change of spot position in the microcavity combined with the fixed fluid force, the precise control of direction and cell-pair rotations Angle is achieved for the first time under the drive of a single fiber, providing a new idea for the field of cell-pair controllable rotation. In the future, this technology will be used to carry out specific research work on beam mode coupling, polarized cell migration, and the predictive effect of studying tissue fluidity and connection dynamics on cancer [29], [30].
